# Schizotypy and personality profiles of Cluster A in a group of schizophrenic patients and their siblings

**DOI:** 10.1186/1471-244X-13-245

**Published:** 2013-10-04

**Authors:** Maria Chiara Torti, Antonino Buzzanca, Chiara Squarcione, Carla Salerno, Alessia Mirigliani, Fabio Di Fabio, Massimo Biondi

**Affiliations:** 1 Department of Neurology and Psychiatry, Sapienza University of Rome, Viale dell’Università 30, Rome 00185, Italy; 2 Department of Psychiatry, Sapienza University of Rome, Rome, Italy; 3 Department of Psychology, Sapienza University of Rome, Rome, Italy; 4 Department of Neuroscience, Sapienza University of Rome, Rome, Italy

**Keywords:** Schizotypy, Schizophrenia, SWAP-200, Personality disorders, First-degree relatives, Siblings

## Abstract

**Background:**

Schizotypy, or the set of personality traits related to schizophrenia, is considered an endophenotypic manifestation that is more represented in first-degree relatives of patients with schizophrenia than in the general population. The assessment of schizotypy is primarily based on self-reports, and for this reason it presents several limitations. In order to assess schizotypy, this study proposes a diagnostic instrument based on clinical reports.

**Methods:**

A sample of 66 subjects, composed of 25 outpatients with schizophrenia, 18 siblings of these patients and 23 healthy controls, was subjected to the personality assessment test SWAP-200 by trained clinical interviewers. To test the hypothesis of the difference between the profiles of the Personality Disorders within the schizophrenia spectrum, a Multivariate Analysis of Variance and subsequent planned comparisons were conducted.

**Results:**

Patients with schizophrenia scored higher than both their siblings and the controls on all SWAP-200 scales; their siblings, compared to the healthy controls, showed significant statistical differences, with higher mean scores for paranoid (F_(1,63)_ = 7.02; p = 0.01), schizoid (F_(1,63)_ = 6.56; p = 0.013) and schizotypal (F_(1,63)_ = 6.47; p = 0.013) traits (PD T scores of Cluster A and Q-factor scores for the schizoid scale [F_(1,63)_ = 6.47; p = 0.013]).

**Conclusions:**

Consistent with previous data, first-degree relatives of patients with schizophrenia scored higher on schizophrenia-related personality traits than a general population comparison sample. SWAP-200, as an alternative diagnostic instrument to self-report measures, is able to reveal the higher prevalence of schizotypal traits in siblings of patients with schizophrenia, suggesting its possible use as a complementary instrument for the assessment of schizophrenia.

## Background

Evidence from adoption, twin, and family studies has shown the importance of genetics in the aetiology of psychosis [1,2].

Data from these studies suggest that genetic vulnerability to developing schizophrenia is present in first-degree relatives of patients with schizophrenia [3], in whom there is a greater genetic risk for psychotic transition than in the general population. Furthermore, this genetic liability can produce “schizophrenia-like” traits (e.g., suspiciousness, eccentricity, social isolation/withdrawal) in these family members, even in the absence of an overt psychotic disorder [4].

The identification of these subthreshold symptoms that genetically correlate with the risk of schizophrenia, which we may term as its endophenotype [1], would be extremely informative for a better understanding of the underlying mechanisms of the illness process of schizophrenia spectrum disorders, as well as for the detection of the involved genes (i.e. “endophenotype strategy” [5]). Schizophrenia-related personality traits are part of this endophenotype [6].

Indeed, it is known that some psychometric instrument-assessed personality traits are expressed under partial genetic control; an example is the evidence of heritability for the standard MMPI scales, estimated between 0.26 and 0.61 (mean = 43.4) [7].

These personality traits, which are more prevalent among the relatives of patients with schizophrenia, constitute the overall expression of the concept of “schizotypy” [8,9].

According to Meehl [10,11], these subclinical schizophrenia-like characteristics, when observed in relatives of psychotic subjects, represent manifestations of the interaction between an inherited “integrative neural defect” predisposing to schizophrenia, termed “schizotaxia”, and the environmental experience of that particular individual. The typical outcome of this interaction is a specific personality organization, which expresses a proneness to develop psychosis, characterized by traits previously described by Kraepelin [12] and Bleuler [13], i.e., interpersonal aversiveness, anhedonia, ambivalence, and cognitive slippage. After Rado [14], Meehl indicated this syndrome as “schizotypy” and the affected individuals as “schizotypal”. He theorized that schizotaxic individuals will manifest schizotypy on a dynamic continuum of increasing severity, which ranges from relative psychological health to full-blown schizophrenia, passing through subclinical degrees of deviance and overt personality disorders. According to this dimensional perspective of Meehl’s schizotaxia-schizotypy paradigm, although a subgroup of schizotaxics can develop schizophrenia, schizotypy is the most typical manifestation of predisposition to the inherited schizophrenia.

Subsequent investigations have shown that symptoms of Schizotypal Personality Disorder reflect increasing degrees of manifestation of schizotypy, in the sense of a “latent personality organization” in schizotaxic individuals [11,14]. Moreover, there is evidence that a detectable Schizotypal Personality Disorder may be particularly useful for enhancing detection of genes contributing to the risk of schizophrenia [15]. These observations have confirmed that patients with Schizotypal Personality Disorder often have a family history of schizophrenia, and that children of mothers with schizophrenia are more likely to develop schizophrenia or schizotypal disorder.

In fact, although several Personality Disorders have been associated with susceptibility to schizophrenia, such as Paranoid Personality Disorder [16-18], Schizoid Personality Disorder and Avoidant Personality Disorder [17], the one most often related to schizophrenia is Schizotypal Personality Disorder [15,16,19-21].

Schizotypal Personality Disorder, according to DSM-IV-TR criteria, is hereditary, and has an estimated incidence between 4.2% and 14.6% [18,22] among adult, non-psychotic first-degree relatives of patients with schizophrenia, compared to 2-3% of the general population [23].

Therefore, since research has indicated schizotypy as a genetic pool from which cases of schizophrenia may arise, a wide range of questionnaires has been developed with the aim of psychometrically detecting people prone to psychosis (see, among others, the positive and negative scales developed by the research team of the University of Wisconsin [24,25], the Schizophrenia Proneness Scale of the MMPI-2 (SzP) [26] and the Schizotypal Personality Questionnaire (SPQ) [27]). However, the tools currently available that enable clinicians to assess schizotypy are based on a format of direct questioning that may be inappropriate. Moreover, some of these tools are also designed specifically to meet the current DSM-IV categories and criteria, limiting their usefulness in making significant revisions to these criteria. Furthermore, as these assessments of schizotypy take a long time and can only be performed by qualified and experienced interviewers, researchers have often used self-report measures as indicators of the risk of developing schizophrenia or psychosis.

Self-report measures, with a few exceptions, proved to be less effective than interviews in distinguishing relatives of patients with schizophrenia from healthy control subjects [28,29]. Several explanations for these findings have been proposed. Some studies have shown a defensive attitude by relatives in answering self-report questions assessing schizotypy, so to deny psychopathology [30,31]. In our opinion, it is necessary to consider this defensive attitude towards schizotypy questionnaires, in order to avoid diagnostic bias.

Compared to interview-based evaluations, self-report questionnaires are less sensitive to specific schizotypal traits in relatives of patients, especially social skills deficits and, probably, odd behavior. Moreover, most self-report assessments evaluate only a few aspects of schizotypy, not referring specifically to those characteristics that family studies have found to be prevalent in relatives of patients with schizophrenia. Finally, these self-report tools do not evaluate the multidimensional aspects of schizotypy or all DSM-IV criteria [28].

These concerns suggest the need for alternative solutions to assess schizotypy.

This work adds to several studies that have previously investigated schizotypal personality traits, conceived as subthreshold manifestations, genetically related to schizophrenia, in healthy relatives of patients with schizophrenia [32-34]; however, the originality of this study lies in the fact that a different perspective is proposed, i.e., the direct observation by experienced clinicians, rather than self-report tools. Therefore, the instrument for personality assessment chosen for the present study is the Shedler-Westen Assessment Procedure (SWAP-200), which differs from other personality and personality disorder instruments in that it was designed for use by expert clinical raters. Thus, SWAP-200 is completed by mental health professionals, typically licensed psychiatrists or psychologists, and not by patients or clients.

The aim of this study is to compare personality traits of patients with schizophrenia, non-psychotic siblings of these patients and healthy controls, using SWAP-200, in order to:

1) Evaluate personality traits related to Cluster A in non-psychotic siblings of included patients.

2) In particular, target the continuum of liability below the threshold of psychosis by evaluating whether mean scores on the scales related to these traits are found in siblings of patients midway between the scores of patients and those of healthy controls (lower than the scores of patients but higher than those of the controls), and if the observed differences have statistical significance.

## Methods

### Ethics

The study was approved by the Ethics Committee for Medical Research (reference number 2333/09.02.2012; prot. 104/12), Policlinico Umberto I° Hospital, Rome, Italy.

### Assessment instruments

The diagnostic test used in this study is the Shedler-Westen Assessment Procedure 200, or SWAP-200 [35-38], a personality assessment instrument that provides clinicians with a detailed and thorough description of the personality of the evaluated subjects, with a systematic and quantifiable approach, designed to maximize both clinical relevance and psychometric precision.

The research assessment SWAP-200 is based on the Q-sort psychometric method [39], using a set of descriptive items (Q-set) that require clinicians to arrange them into a fixed distribution (in a defined number of groups).

This diagnostic procedure ensures diagnosis of Personality Disorders (PD) both categorically and dimensionally, providing PD scores specifically referring to the DSM-IV criteria, and Q scores (Q sort) empirically derived and referring to the Psychodynamic Diagnostic Manual (PDM) [40].

An experienced clinician, usually a clinical psychologist or a psychiatrist, assigns a score from 0 to 7 for each of the 200 proposed personality-descriptive items; the score identifies each item in a range from “not describing the patient” to “highly describing the patient”. Descriptions of personality for each patient using SWAP-200 seem to be similar to those of the Minnesota Multiphasic Personality Inventory (MMPI), in particular, regarding the matches between the personality profile of the patient and the profile of the control group, except that these profiles are not self-reports (as they are in MMPI) but rely on the judgments of a clinician-observer.

SWAP-200 does not assume that patients can report their maladaptive personality traits by themselves. Rather, it presumes that a skilled clinical interviewer, through a systematic clinical interview, or through longitudinally knowing the patient during the period of professional contact, can identify these pathological traits.

This ensures a systematic and objective assessment of the patient, through a diagnostic instrument that evaluates both the syndromal aspects and global functioning of the patient in the context of his life.

Regarding the psychometric properties of SWAP-200, we mention an illustrative study examining the reliability and validity of trait scores derived from SWAP-200 via factor analysis [41].

The study assessed the interrater reliability and validity of the 12 SWAP-200 trait scale scores, which are comparable to trait dimensions assessed by self-report measures.

Interrater reliability by interview was high, with median correlations between independent interviewers of 0.82. Convergent and discriminant validity (assessed by cross-informant correlations between the independent interviewers and treating clinician) were also strong, with a median convergent validity coefficient (on the diagonal) of *r* = 0.66 and a desirably low median discriminant validity coefficient (off the diagonal) of *r* = –0.06.

In the present study, to verify the results derived by SWAP-200, a direct comparison between these observer-rated data and a self-report measure was made.

This self-report assessment instrument is TALEIA 400A (Test for AxiaL Evaluation and Interview for clinical, personnel, and guidance Applications), a measure developed in Italy by Boncori and Coworkers [42] and translated into several languages. TALEIA quantifies both clinical and personality disorders. Its 400 items refer mainly to specific everyday situations. Subjects are required to report the frequency of each behavioral occurrence on a four-level Likert-type scale (always, often, sometimes, and never). Psychometric research on the English translation of this instrument is in progress in the U. S. [43]. TALEIA consists of 21 scales (18 clinical scales based on the diagnostic criteria of DSM-IV-TR and 3 validity scales). Several studies have demonstrated the validity of this test in clinical assessment [42-45]. Furthermore, TALEIA’s scores were compared with scores of well-known psychopathological measures such as MMPI and MMPI-2. The findings of these comparison studies demonstrated Pearson correlation coefficients beyond the significance level of p = 0.01.

In our research, we used the Cluster A Personality Disorders scales Paranoid (PP), Schizoid (PSK) and Schizotypal (PSKT).

### Participants

The participant pool for this preliminary study was composed of 66 subjects: 25 outpatients with schizophrenia (14 females, 11 males, mean age: 36.28 years ± 9.43 SD), 18 siblings of the patients with schizophrenia (6 female, 12 males, mean age: 36.78 years ± 10.87 SD) and 23 healthy controls (12 females, 11 males, mean age: 32.26 years ± 7.61 SD).

All participants were recruited in the context of a broader research project on endophenotypes, currently ongoing at the Department of Neurology and Psychiatry of Umberto I Hospital in Rome, Italy, and filled out an informed consent form after receiving a full description of the research project.

For several reasons, first-degree relatives of patients with schizophrenia can differ from each other, in particular in age at recruitment and in the risk of developing schizophrenia (relative risk: parents 5%; siblings and offspring 10%) [16]; so, in order to remove these differences, only the siblings of the schizophrenic patients enrolled in this study were included. Exclusion criteria considered were neurological disorders and drug and alcohol dependence.

Diagnosis of schizophrenia, in the patient group, was made by three expert psychiatrists (MCT, FDF, AM), according to DSM-IV criteria [46]. All patients, our outpatient-service clients for at least two years, were being treated with second-generation antipsychotic drugs at the time of the study and were in a phase of psychopathological stability, with minimal or no florid symptoms. Their social functioning was sufficiently preserved to allow an effective interaction with the clinicians.

Siblings of patients with schizophrenia and healthy controls did not differ on socio-demographic variables, and were screened with the SCID-I/NP (Non-Patient Edition) [47] to exclude psychotic disorders.

### Procedure

All participants were interviewed by three psychiatrists (MCT, AM, FDF) and a clinical psychologist (AB).

These four raters, all with at least three years of clinical experience after their specialization degrees, underwent training in the use of SWAP-200; at the conclusion of the training, a full recorded semi-structured interview of an unknown patient was submitted to them. Raters were asked to provide scores for every item. The analysis of the interrater-reliability was assessed by a bivariate correlation obtaining an acceptable index of agreement (*r* = 0.74).

For siblings and healthy controls, socio-demographic data like gender, age, education and marital status were collected through an interview at the beginning of the study. Patient data were derived through a review of their medical records. Healthy participants were evaluated with SCID-I/NP. All subjects filled out the TALEIA questionnaire. Thereafter, all participants were evaluated with SWAP-200 through at least three clinical interviews lasting roughly two hours each.

In these interviews, as in typical clinical practice, clinicians look to subjects’ narratives about their daily lives and to observed behavior in interaction with the interviewer. In particular, the survey focuses on their symptoms, their education and work history, and their relationships, requiring examples of emotionally salient experiences. From these data, the clinicians make judgments about the ways the subjects characteristically think, feel, view self and others, regulate impulses and behave in significant relationships, consistently assigning a score to the items.

This suggests that the extensive knowledge of the subject SWAP-200 requires allows the clinician to immediately detect the presence of psychopathology, preventing the execution of the interview by interviewers blind to diagnosis. For this reason, in the present work as in previous studies, this procedure was carried out as an open label study.

### Statistical analysis

Data were processed and analyzed using SPSS for Windows, version 19.0.

To test the hypothesis that PD scores of SWAP-200 in at least two groups of subjects were significantly different, the Multivariate Analysis of Variance (MANOVA) was used. This kind of analysis consists of an independent variable with three levels, which are the Patient Group, the Siblings Group and the Healthy Controls Group, and of four dependent variables, namely, the Cluster A Personality Disorders scale and the High Functioning scale of SWAP-200. The analysis was repeated using the same independent variable and the scores of two Q-factor scales of Personality Disorders of schizophrenic spectrum of SWAP-200.

The analysis of the effects was assessed by Wilks’ Lamba, Pillai’s Trace, Hotelling’s Trace and Roy’s Root.

Then, the results of the multivariate planned comparisons between the Patients group *vs.* the Siblings group, and the Siblings group *vs.* the Healthy Control group were analyzed, as were the effects of the univariate comparisons between the same groups.

For the analysis of the multivariate and univariate effects and of the planned comparisons, the significance level was set at p < 0.05.

To correlate the scales of SWAP-200 with the scales of TALEIA, bivariate correlations with the Pearson correlation coefficient were made.

## Results

Before the application of multivariate tests, assumptions of normality of the distributions of the independent variables considered in the analysis were evaluated; the indices of skewness and kurtosis ranged between 1 and -1. Due to this, the values in this range indicate that non-normality is not a source of serious distortions [48]. Levene’s Test of Equality of Error variances was not significant for any independent variable, thus verifying the assumption of homoschedasticity of variances.

To test the difference between groups considered in PD scale for the Cluster A of SWAP-200 multivariate analysis of variance was performed, and showed that the Groups Effect was significant, (Wilk’s Lambda_(4,60)_ < .001; p < 0.001): this result underlines that at least two groups are different in the scores for Personality Disorders and High Functioning, considered as a whole. Pursuing the analysis of the univariate tests for the effects between subjects, all the considered scales showed statistical significance at the p < 0.001 level. In investigating the results of the multivariate planned comparisons, the first (Patients *vs*. Siblings) was significant (Wilk’s Lambda_(4,60)_ = 0.389; p < 0.001); the analysis of the results of univariate planned comparisons for the Cluster A Personality Disorders scales showed significant differences (Pa F_(1,63)_ = 7.224 p = 0.009; Sch F_(1,63)_ = 58.31 p < 0.001; Szt F_(1,63)_ = 97.721 p < 0.001), similarly to the High Functioning scale (HF) (F_(1,63)_ =63.28).

The multivariate comparison of Siblings *vs*. Healthy Controls was significant (Wilk’s Lambda_(4,60)_ = 0.848; p = 0.039); in deepening the analysis of the results of univariate planned comparisons, it showed statistically significant differences (PD Para, F_(1,63)_ = 7.02 p = 0.01; PD Sch, F_(1,63)_ = 6.56 p = 0.013; PD Szt, F_(1,63)_ = 6.1 p = 0.016; HF, F_(1,63)_ = 5.30).

The statistical hypothesis test of the difference between groups considered in Q-factor scale of SWAP-200 was significant (Wilk’s Lambda_(2,62)_ = 0.11; p < 0.001); this meant that at least two groups considered in the Schizophrenia Spectrum scale of Q-factor scale of SWAP-200 test were different.

Considering the results of the univariate comparisons for effects between subjects, it was noted that the difference was statistically significant for the two Q-factor scales Schizoid (F_(2,63)_ = 73.94; p < 0.001) and Paranoid (F_(2,63)_ = 3.22; p = 0.046). The first multivariate planned comparison (Patients *vs*. Siblings) was significant (Wilk’s Lambda_(2,62)_ = 0.465; p < 0.001). The analysis of the univariate results of the first planned comparison showed significant differences for the Q-factor scale Schizoid (F_(1,63)_ = 69.04; p < 0.001), but not for Paranoid (F_(1,63)_ = 0.773; p = 0.383). Also, the multivariate comparison between Siblings *vs*. Healthy Controls was significant (Wilk’s Lambda_(2,62)_ = 0.868; p = 0.013). The analysis of the univariate planned comparisons showed a significant difference only for the Q-factor scale Schizoid (F_(1,63)_ = 6.47; p = 0.013) (Q factor Paranoid F_(1,63)_ = 2.11; p = 0.151).

**Figure 1:**
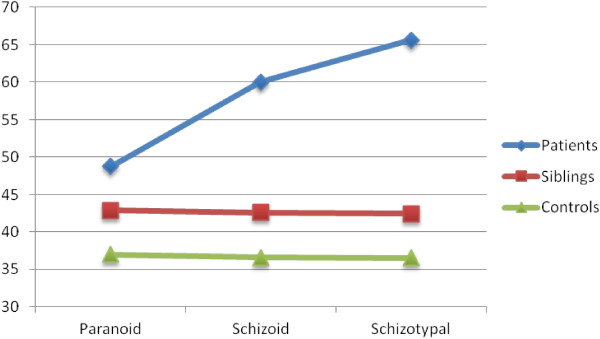
Estimated marginal means for the scores of the PD T scales of cluster A in the three groups.

As can be seen from Table 1, regarding the scores of the PD T scales considered in our analysis, the Patient group scored significantly higher than the Siblings group on the Personality Disorders scales in the first comparison (Patients *vs*. Siblings), while on the High Functioning scale the Siblings group scored higher than the Patients group; in the second planned comparison of PD T scale scores (Siblings *vs*. Healthy Controls), the Siblings group scored significantly higher on the Personality Disorder scale than Healthy Controls, while on the High Functioning scale, the Healthy Control group scored higher than the Siblings group. The estimated marginal means for PD T scale scores related to Cluster A Personality Disorders for all three groups are summarized in Figure 1.

**Table 1:** Estimated marginal means for PD scales

**Dependent variable**	**Group**	**Mean**	**Std. Error**	**95% Confidence interval**	
				**Lower**	**Upper**
				**bound**	**bound**
PD T Para	Patients	48.772	1.414	45.945	51.599
	Siblings	42.896	1.667	39.565	46.227
	Controls	37.000	1.475	34.053	39.947
PD T Sch	Patients	60.038	1.479	57.084	62.993
	Siblings	42.589	1.742	39.107	46.071
	Controls	36.630	1.541	33.550	39.711
PD T Szt	Patients	65.634	1.517	62.603	68.665
	Siblings	42.459	1.788	38.887	46.031
	Controls	36.566	1.581	33.406	39.726
PD T HF	Patients	46.722	1.448	43.828	49.616
	Siblings	64.527	1.707	61.116	67.937
	Controls	69.766	1.510	66.748	72.783

For the analysis of the planned comparisons of Q-factor scales considered in our study, the Patient group scored higher than the Siblings group on the Schizoid Q-factor scale (estimated marginal means for the first comparison, i.e., Patients *vs*. Siblings), while in the second planned comparison (Siblings *vs*. Healthy Controls), the Siblings group scored significantly higher than the Healthy Control group on the same scale (Table 2).

**Table 2:** Estimate marginal means for Q factor scales

**Dependent variable**	**Group**	**Mean**	**Std. Error**	**95% Confidence interval**	
				**Lower**	**Upper**
				**bound**	**bound**
Q T Sc	Patients	64.103	1.571	60.964	67.241
	Siblings	43.933	1.851	40.235	47.632
	Controls	37.647	1.637	34.375	40.919
Q T Pa	Patients	46.346	1.401	43.547	49.145
	Siblings	44.443	1.651	41.144	47.741
	Controls	41.240	1.460	38.321	44.158

As expected (and shown in Table 3), the scales of SWAP-200 and TALEIA for the same constructs showed positive and statistically significant correlations (Paranoid N = 66, *r* = 0.531 p < 0.01; Schizoid N = 66, *r* = 0.401 p < 0.05; Schizotypal N = 66, *r* = 0.549 p < 0.01), highlighting a notable concurrent validity of SWAP-200.

**Table 3:** Matrix of bivariate correlations between the considered scales of SWAP-200 and the cluster A scales of TALEIA

		**PP**	**PSK**	**PSKT**
PD T Para	Pearson’s *r*	.531^**^	.271	.396^*^
	Sig. (2-tailed)	.003	.140	.030
	N	66	66	66
PD T Sch	Pearson’s *r*	.427^*^	.401^*^	.467^**^
	Sig. (2-tailed)	.019	.025	.009
	N	66	66	66
PD T Szt	Pearson’s *r*	.523^**^	.446^*^	.549^**^
	Sig. (2-tailed)	.003	.012	.002
	N	66	66	66
Q T Sc	Pearson’s *r*	.426^*^	.390^*^	.467^**^
	Sig. (2-tailed)	.019	.030	.009
	N	30	31	30
Q T Pa	Pearson’s *r*	.466^**^	.206	.318
	Sig. (2-tailed)	.010	.266	.087
	N	66	66	66

**The correlation is significant at p 0.01 (2-tailed).

*The correlation is significant at p 0.05 (2-tailed).

Notes:

*for PD scales of SWAP-200*: Para = Paranoid; Sch = Schizoid; Szt = Schizotypal; HF = High Functioning.

*for Q factor scales of SWAP-200*: Sc = Schizoid; Pa = Paranoid.

*for Cluster A scales of TALEIA*: PP = Paranoid; PSK = Schizoid; PSKT = Schizotypal.

The significant correlations between the Cluster A scales of TALEIA and the not homonymous PD T scales can be explained by the fact that some indicators of the Cluster A scales are overlapped.

The Q-factor scale Schizoid showed positive correlations with all the considered scales of TALEIA (Paranoid N = 66, *r* = 0.426 p < 0.05; Schizoid N = 66, *r* = 0.390 p < 0.05; Schizotypal N = 66, r = 0.467 p < 0.01) but the Q-factor scale Paranoid showed positive correlations only with the Paranoid scale of TALEIA (N = 66, *r* = 0.466 p < 0.01).

The Q-factor Schizoid includes the DSM-IV diagnosis of Schizotypal, Schizoid and Avoidant Personality Disorder, recognized as difficult to distinguish.

The results of the Q-analysis from previous studies by the authors of the methodology SWAP denote a lack of clear boundary lines between these three categories, confirmed by the results from the analysis of correlations in our research.

This element could explain the first set of significant correlations between the scales of TALEIA and the Q-factor Schizoid.

## Discussion and conclusion

As an alternative to self-reports, we used SWAP-200 in this preliminary study to assess schizotypy.

Our preliminary results are consistent with previous data supporting the higher prevalence of schizotypal traits in first-degree relatives of patients with schizophrenia compared to the general population [15,19-21,34].

In particular, analyzing our data we may observe that:

1. *In the items that meet the criteria for Cluster A Personality Disorders of DSM-IV, siblings of patients with schizophrenia show significant differences compared to healthy controls, with higher scores for paranoid, schizoid and schizotypal traits.*

Concerning the PD T scores, which are based on the DSM-IV Cluster A of Personality Disorders, it is possible to note that the scores of the scales considered for the Siblings group are intermediate between the scores of the other two groups, showing statistically significant differences not only compared to the Patient group, as expected, but also compared to the Healthy Control group. This element is prominent because the scores for paranoid, schizoid and schizotypal traits of siblings are significantly lower than the scores for the same traits of patients with schizophrenia, but are significantly higher than those obtained by the healthy controls.

2. *In the items that meet criteria for the P Axis of the PDM for the evaluation of personality patterns and Personality Disorders, these significant differences are reconfirmed for the schizoid scale.*

These data were also confirmed by the analysis of Q-scores, hence showing validity also in the context of a different taxonomy.

Regarding the Q-factor scores on the schizoid scale, our analysis revealed a tendency of the Siblings group to score midway between the Patient group and the Healthy Control group. Compared to patients with schizophrenia, siblings scored significantly lower on schizoid scale, but compared to the Healthy Control group, the Siblings group confirmed the trend to score significantly higher. For the Q-factor Paranoid scale, the differences between the three groups were not significant, but the usual trend of the Siblings group to score higher than Healthy Controls and lower than Patients, was confirmed.

The analysis of correlations with the self-report tool confirmed reliability and validated what the external raters observed.

Based on data presented in this study, we may conclude that SWAP-200, similarly to self-report measures, is able to accurately highlight the presence of schizotypal traits in first-degree relatives of patients with schizophrenia, according to both the DSM-IV categorical diagnostic approach and the dimensional model proposed by PDM.

Clinicians have often proposed to replace the current diagnostic assessment for Personality Disorders with dimensional models [35]. These proposed models derive their data mainly from self-report issues. A different approach is to derive personality dimensions from data provided by expert clinicians.

In addition to developing a diagnostic tool, the authors of the methodology SWAP have tried to introduce a new way of understanding personality assessment: they have combined categorical and descriptive logic to a dimensional approach, integrating scientific rigor with clinical utility.

In a dimensional approach, the assessment is not carried out by defining the presence or absence of specific traits, but by considering how strongly they arise or come close to the diagnostic prototype.

In this sense, SWAP-200 is particularly suitable to detect subthreshold manifestations of schizotypy and for tracking disease susceptibility in non-clinical genetic carriers.

Besides the implication for the validity of the schizophrenia-spectrum paradigm, our results suggest the clinical importance of a dimensional diagnosis for refining the identification of spectrum phenotypes both in genetic research and in early detection.

Literature has already confirmed the reliability and the validity of SWAP-200 as a predictor of maladaptive traits, such as suicide attempts and previous psychiatric hospitalizations, and of global functioning, clinical diagnosis, pathological development variables, and medical history [49,50], hence contributing to the collection of information consistent with the patient’s development, outcome, and global functioning.

Based on these results, we propose SWAP-200 also as a diagnostic instrument to detect schizotypal personality traits, as well as for a complete evaluation of the global profile of the patient.

### Limitations of the study

This study has two key limitations:

First, selection “bias” may have resulted from the possibility that the siblings willing to enroll in studies of this type have personality traits associated with better functioning and volunteer motivation.

The second limitation is that the sample size is relatively small (less than 30 subjects per group).

Future research in larger samples, preferably carried out with both SWAP-200 and other self-rated tools (i.e. SCID II; 16PF; MCMI-III), may address the above limitation.

## Competing interests

The authors declare that they have no competing interests.

## Authors’ contributions

MCT carried out the study and drafted the first version of the manuscript. AB analyzed the data. CS, CS, AM and FDF participated in the design of the study and helped to draft the manuscript. MB conceived of the study and helped to draft the manuscript. All authors read and approved the final manuscript.

## Authors’ information

MCT and AM are psychiatrists and PhD students at ‘Sapienza’ University of Rome. AB is psychologist and postdoctoral fellow at the same University. CS and CS are undergraduate students at the Faculty of Medicine and Surgery in the same University. FDF is a psychiatrist at Policlinico Umberto I° Hospital, Rome. MB is Full Professor of Psychiatry at ‘Sapienza’ University of Rome and Chief of the Psychiatry and Mental Health Area, Policlinico Umberto I° Hospital, Rome.

## Pre-publication history

The pre-publication history for this paper can be accessed here:

http://www.biomedcentral.com/1471-244X/13/245/prepub
